# Patient Willingness to Dispose of Leftover Opioids After Surgery: A Mixed Methods Study

**DOI:** 10.1097/AS9.0000000000000223

**Published:** 2022-12-07

**Authors:** Phoebe Draper, Josh Bleicher, Jaqueline K. Kobayashi, Elizabeth L. Stauder, Gregory J. Stoddard, Jordan E. Johnson, Jessica N. Cohan, Kimberly A. Kaphingst, Alex H. S. Harris, Lyen C. Huang

**Affiliations:** From the * Ronald O. Perelman Department of Emergency Medicine, New York University Langone Health, New York City, NY; † Department of Surgery, University of Utah, Salt Lake City, UT; ‡ Department of Internal Medicine, University of Utah, Salt Lake City, UT; § Huntsman Cancer Institute, Salt Lake City, UT; ∥ Department of Communication, University of Utah, Salt Lake City, UT; ¶ Department of Surgery, Stanford University, Stanford, CA; # Center for Innovation to Implementation, VA Palo Alto Healthcare System, Palo Alto, CA.

**Keywords:** acute pain, analgesic, financial incentive, medical waste disposal, mixed methods, opioid stewardship, opioids, prescription drug misuse, surgery

## Abstract

We examined how convenience and financial incentives influence patient willingness to dispose of leftover prescription opioids after surgery. We also identified additional barriers and facilitators to disposal.

**Background:**

In the United States, up to 70% of surgical patients are prescribed opioids and up to 92% will have leftover tablets. Most do not dispose of leftover opioids, increasing the risk for opioid-related harm. Current interventions promoting opioid disposal have shown mixed success.

**Methods:**

We conducted a mixed methods study using a standard gamble survey and semi-structured interviews. Participants estimated willingness to dispose in 16 scenarios with varying convenience (time requirements of <5, 15, 30, and 60 minutes) and financial incentives ($0, $5, $25, $50). We estimated the likelihood of disposal using a multivariable mixed effects modified Poisson regression model. Semi-structured interviews explored how convenience, financial incentives, and other barriers and facilitators influenced decisions to dispose.

**Results:**

Fifty-five participants were surveyed and 42 were interviewed. Most were willing to dispose when the time required was <15 minutes. Few were willing to dispose if the process required 60 minutes, although a $50 financial incentive increased rates from 9% to 36%. Anxiety about future pain, opioid scarcity, recreational use, family safety, moral beliefs, addiction, theft, and environmental harm also influenced decision-making.

**Conclusions:**

Interventions promoting opioid disposal should focus on convenience, but the selective use of financial incentives can be effective. Tailoring interventions to individual barriers and facilitators could also increase disposal rates.

## INTRODUCTION

In the United States, up to 70% of patients are prescribed opioids after surgery^1,2^ and up to 92% of these patients will have leftover tablets.^3–7^ As many as 91% of post-surgical patients do not dispose of leftover opioids, demonstrating an area in need of improvement.^5,8,9^ Leftover prescription opioids are often stored insecurely, placing patients and communities at risk for opioid-related harm.^5,6,10,11^ Between 1999 and 2019, 247,000 people died in the United States from prescription opioid overdoses.^12^ In 2019, 28% of drug overdose deaths in the United States involved prescription opioids.^13,14^ Recognizing improved opioid disposal as an area of need, the Stanford-Lancet Commission on the North American Opioid Crisis recently called for raising the quality of excess opioid disposal programs to foster healthier environments and reduce the incidence of addiction.^15^

The Drug Enforcement Administration launched the first nationwide prescription take-back program in 2010.^16^ Since then, drop boxes,^17^ community take-back events,^18^ home disposal bags,^19–22^ patient education,^23–25^ and financial incentives^26,27^ have been used to promote opioid disposal. These interventions have had mixed success. For example, take-back events often cite impressive quantities of returned medications, but most are not controlled substances like opioids.^17^ Interventions combining convenient disposal options such as home disposal bags or clinic-based drop boxes with patient education have shown promise in some studies (disposal rates of 55%–83%),^7,19–21^ but disappointing results in others (10%–14%).^22^ One survey found 84% of patients would be more likely to use a drug take-back service if they were offered a small financial incentive, but a Veterans Affairs pilot study found only 29% of rural veterans returned leftover opioids despite being offered $5 per returned tablet (max $50).^26,27^ Why certain interventions have been more successful than others remains unclear.^8,9^ Our group and others previously identified convenience, financial incentives, disposal method awareness, low perceived risk of nondisposal, mistrust of law enforcement, future use, and forgetfulness as facilitators and barriers to disposal.^22,28–31^ However, how these factors interact with each other to influence willingness to dispose has not been tested.

The primary goal of this study was to understand how the relationship between convenience and financial incentives influences patient willingness to dispose of leftover prescription opioids after surgery. Using a mixed methods approach, we used a standard gamble model to examine patients’ willingness to participate in a theoretical opioid buyback intervention and how participation is influenced by 2 overlapping variables: convenience (measured by time required for disposal) and financial incentive (measured by different monetary amounts).^32^ In parallel, we conducted qualitative interviews to examine how other barriers and facilitators further influence involvement in the intervention. Understanding how convenience, financial incentives, and other factors influence patients’ decisions to dispose of opioids will guide the development and refinement of successful prescription opioid disposal interventions.

## METHODS

### Study Design and Participant Recruitment

We used a mixed methods design, collecting quantitative data through standard gamble surveys in parallel with qualitative interviews, to better understand patient decision-making.^33^ We invited adult patients (age > 18 years) who presented to general surgery clinics for preoperative consultation at a single academic medical center between January 28, 2021 and March 29, 2021 to participate. Patients did not have to schedule an operation to participate. We a priori determined 42 participants were required to achieve 90% power, using a 2-sided alpha 0.05 comparison, to detect an opioid disposal willingness greater than 50%. Written informed consent was provided by all participants, and this study was approved by the University of Utah Institutional Review Board (IRB 00139736).

### Data Collection

Eligible patients were invited to participate after their clinic appointment. One of 2 members of the research team consented and enrolled participants, then guided them through a survey followed by an approximately 15-minute interview. For the survey, participants completed a matrix based on standard gamble methodology^32^ to indicate willingness to dispose of leftover opioids under various scenarios. Four disposal durations (<5, 15, 30, and 60 minutes) were paired with 4 financial incentives ($0, $5, $25, and $50), creating 16 distinct scenarios. The scenarios were presented as a matrix, so participants could recognize the scenarios involved a trade-off between convenience and financial incentives. Participants indicated on a Likert scale from 1 (very unlikely) to 5 (very likely) their likelihood of disposing leftover opioids. They were allowed to adjust their responses as they considered all scenarios. We capped our financial incentive at $50, as greater amounts are likely not feasible in any large-scale disposal intervention. The survey also asked participants to report their age, gender, race, ethnicity, household income, prior receipt of prescription opioids, prior use of opioids, household income, home zip code, access to a private vehicle, current smoking or vaping or tobacco use, and medical history (eg, mental health disorders, substance abuse, opioid abuse). We used the Federal Office of Rural Health Policy Data Files to categorize patients as rural or urban.^34^ For the qualitative portion, participants were asked open-ended questions such as “What do you know about opioid disposal programs?” “What factors did you consider in your decision making?” “What would make you more likely to dispose?” and “What would make you less likely to dispose?”. All participants were asked these core questions, with follow-up questions asked to explore responses in more depth. The survey and interview guide (Supplement 1, http://links.lww.com/AOSO/A186) were developed based on available evidence from prior studies on the determinants of opioid disposal and with assistance from experts in surgery, substance use disorders, perioperative health services research, and qualitative and mixed-methods methodology. The interview guide was further refined through cognitive pilot-testing during the first 10 interviews. Interviews were recorded then transcribed verbatim. COnsolidated criteria for REporting Qualitative research (COREQ) guidelines were followed for the qualitative portion of this study (Supplement 2, http://links.lww.com/AOSO/A186).

### Quantitative Statistical Analysis

For each disposal scenario, we created a binary outcome. Participants who responded with “likely” or “very likely” were coded as “willing to dispose.” The responses “very unlikely,” “unlikely” and “unsure” were coded as “unwilling to dispose.” Unsure was coded as unwilling to dispose, as prior studies suggest patients who are “unsure” are unlikely to dispose.^30^ Although it ultimately comes down to a decision (or action) to dispose or not (a binary outcome), which is how a policy maker needs to see the data, scoring the scenarios on a 5-point Likert scale allows participants to express uncertainty. Specifically, participants could provide a response that varied along a continuum without the pressure of providing an immediate decision, thereby avoiding a response pressure bias leading to guessing. After dichotomizing responses into “willing to dispose” versus “unwilling to dispose,” the data represented 16 binary decisions from each participant. To test for statistically significant scenarios where participants were more likely to dispose than not, we fit univariable and multivariable mixed effects modified Poisson regression models, with robust standard errors, to the binary outcome, using participant identification number as a random effect.^35^ This approach accounted for clustering at the participant-level. The fixed effects predictor variables were convenience (a categorical variable with 4 categories [5, 15, 30, and 60 minutes] modeled using 3 indicator variables), financial incentive (a categorical variable with 4 categories [$0, $5, $25, $50] modeled using 3 indicator variables), and convenience × financial incentive interaction terms. We used post-fit marginal estimation to obtain the 95% confidence intervals associated with willingness to dispose for each scenario. Confidence intervals not crossing the 50% threshold represent a statistically significant scenario (ie, confidence intervals above 50% represent a scenario where participants are significantly more likely to dispose).

We fit univariable models adding 1 patient-level variable as a fixed effect to determine which patient demographic and clinical factors influenced willingness to dispose. We categorized participants as White or non-White, as the sample size precluded further subcategorization. As participants completed the survey with a member of the research team, there was no missing data, and no data imputation was required. Statistical analyses were performed using Stata Version 15.1 (Stata Corp, College Station, TX).

### Qualitative and Mixed Methods Analysis

We analyzed the qualitative data using an inductive analysis (without a predetermined theoretical framework) to allow for barriers and facilitators that may not have been identified in prior studies or frameworks. An initial codebook was created by the research team based on prior research on the determinants of opioid disposal.^22,28–31^ A random 10% sample of the transcribed interviews were reviewed by 2 members of the research team. They used individual interviews as the unit of analysis. After initial coding, the research team met to review discrepancies in coding, revising the codebook as needed. An additional 10% of interviews were then coded with subsequent codebook revision. This process was repeated 3 times and continued until each code was exhaustive and unique with a high inter-rater reliability between coders (Cohen’s kappa value of 0.8 or higher for each code).^36^ Once complete, the remaining interviews were coded independently by a single member of the research team. All investigators analyzed the codes for recurrent themes. We performed quantitative analysis on questions answered by all patients to determine the percentage who held certain views toward opioid disposal. Finally, we sought to explain our survey’s quantitative results through the lens of the qualitative data using triangulation and integration.^37^

## RESULTS

### Participant Demographic Characteristics

We approached 61 eligible patients and 55 completed the survey (response rate 90%). The first 42 participants completed the survey and semi-structured interview. The remaining 13 participants completed the survey only as we had reached thematic saturation with our qualitative data and no further interviews were required. The median age was 53 (interquartile range, 36–63) and 29 (53%) were female (Table 1). Approximately one-third reported a household income <$50,000 and one-quarter reported a household income >$100,000. Fifty-one participants (93%) had access to a private vehicle and 17 (31%) resided in a rural zip code. Most participants had filled an opioid prescription in the past year (n = 32, 58%), 7 (13%) had used opioids regularly in the past year, and 3 (5%) reported a history of opioid abuse. No patient-level demographic or clinical characteristics were significantly associated with disposal willingness (Table 2).

**TABLE 1.** Demographic and Clinical Characteristics of the 55 Participants

Variable	N (%)
Age, median (IQR)	53 (36–63)
Gender	
Female	29 (53)
Male	26 (47)
Race	
White	43 (78)
Black	2 (4)
Native Hawaiian/Pacific Islander	1 (2)
Asian	3 (5)
Other	6 (11)
Ethnicity	
Hispanic	5 (9)
Non-Hispanic	50 (91)
Household income (in USD)	
0–50,000	17 (31)
50,001–100,000	21 (38)
>100,000	15 (27)
Decline to answer/unknown	2 (4)
Access to a private vehicle	51 (93)
Home state	
Idaho	5 (9)
Maryland	1 (2)
Nevada	4 (7)
Utah	41 (75)
Wyoming	4 (7)
Rural home location (by zipcode)	17 (31)
Current smoker, vaper, or tobacco users	9 (16)
History of mental health diagnosis	25 (45)
History of substance abuse	12 (22)
Filled opioid prescription in the past year	32 (58)
Used opioids regularly in the past year	7 (13)
History of opioid abuse	3 (5)

IQR indicates interquartile range; USD, US dollars.

**TABLE 2.** Univariable Analysis of Factors Associated With Participant Willingness to Dispose

Variable	Univariable Model	
	RR (95% CI)	*P*
Age, per 10-year increase	0.91 (0.82–1.02)	0.091
Gender		
Male	0.88 (0.62–1.25)	0.47
White race	1.28 (0.91–1.80)	0.16
Hispanic ethnicity	1.33 (0.99–1.78)	0.06
Household income, per $50,000 increase		
0–50 k	Reference	—
50,001–100 k	1.11 (0.70–1.78)	0.66
>100 k	1.29 (0.84–1.99)	0.25
Access to a vehicle	0.95 (0.50–1.77)	0.86
Rural home location	0.81 (0.52–1.25)	0.34
Current smoker, vaper, or tobacco user	1.34 (0.91–1.98)	0.135
History of mental health diagnosis	0.92 (0.65–1.29)	0.62
History of substance abuse	0.89 (0.55–1.44)	0.63
Filled opioid prescription in the past year	0.81 (0.58–1.13)	0.21
Used opioids regularly in the past year	1.08 (0.56–2.01)	0.81
History of opioid abuse	1.24 (0.88–1.74)	0.21

CI indicates confidence interval; RR, risk ratio.

### The Influence of Convenience and Financial Incentives on Willingness to Dispose

Participants were asked to rate their likelihood of disposing of opioids for 16 scenarios of 4 different time requirements (<5, 15, 30, and 60 minutes) and 4 financial incentives ($0, $5, $25, and $50) (Table 3). Most were willing to dispose in the scenario where convenience was maximized (<5 minutes required to dispose), even without financial incentive ($0 provided for disposal) (n = 34, 62%). If the time required to dispose was <5 minutes, increasing financial incentives to $5, $25, or $50 increased willingness to dispose to 67%, 80%, and 83%, respectively. With maximum inconvenience (60 minutes required to dispose) and no financial incentive, few patients were likely to dispose (n = 5, 9%). Increasing the financial incentive to $5, $25, and $50 increased disposal willingness to 13%, 18%, and 36%, respectively. Ten participants (17%) were unwilling to dispose even with the least time requirement and highest financial incentive (<5 minutes and $50 incentive).

**TABLE 3.** Participants Willing to Dispose of Their Leftover Opioids Based on Tradeoffs Between Convenience and Financial Incentives, Reported As n (%)

Financial Incentive (US Dollars)	Convenience (Time Required to Dispose)			
	<5 minutes	15 minutes	30 minutes	60 minutes
$0	34 (62%)	28 (51%)	12 (22%)	5 (9%)
$5	37 (67%)	30 (55%)	13 (24%)	7 (13%)
$25	44 (80%)	38 (69%)	22 (40%)	10 (18%)
$50	45 (83%)	43 (78%)	29 (53%)	20 (36%)

**FIGURE 1.**
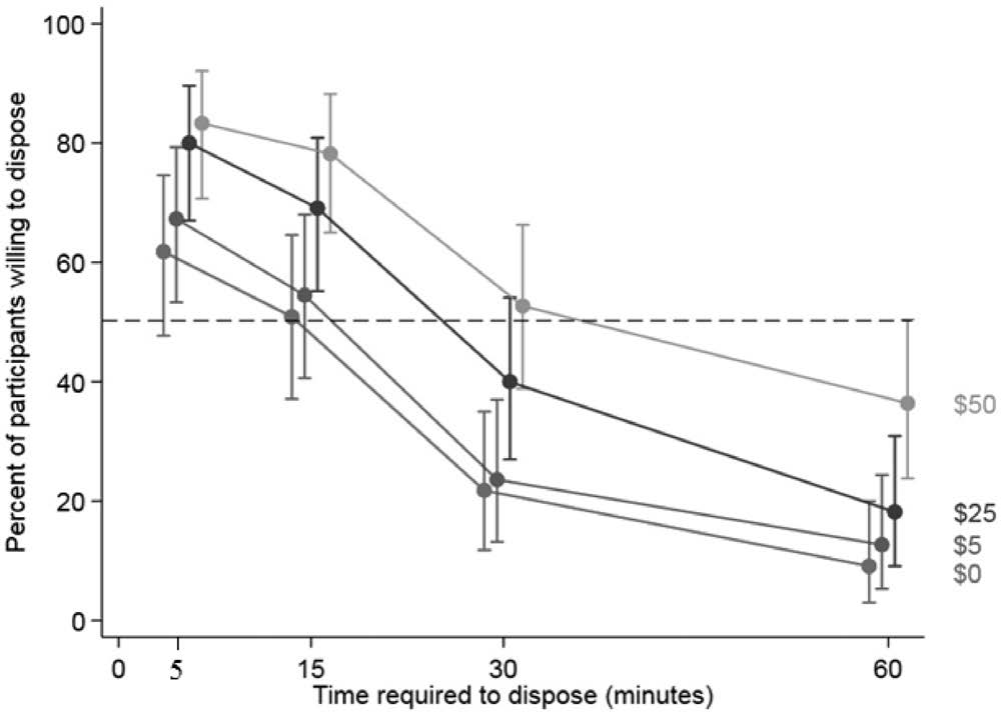
Multivariable modified Poisson regression model* showing the estimated percentage of participants willing to dispose of their leftover opioids for each scenario (vertical axis) for 16 scenarios with tradeoffs between convenience and financial incentives. The horizontal axis represents convenience (the time required to dispose). The vertical bars represent the 95% confidence interval. The dotted line indicates a threshold representing 50% of participants willing to dispose. Confidence intervals not crossing the 50% threshold represent statistically significant scenarios. *Fixed effects predictor variables: convenience, financial incentive, and convenience × financial incentive interaction terms. Random effect: participant identification number.

After using the multivariable Poisson regression model to generate 95% confidence intervals for each scenario, we saw a consistent pattern of decreasing disposal willingness as time required to dispose increased (Fig. 1). Financial incentives increased willingness to dispose but not to the extent as convenience.

### Interview Results

Our qualitative analysis found knowledge of opioid disposal programs fell into 3 categories: “minimal knowledge,” “limited knowledge” (able to describe a method of disposal but describing inaccurate information or using unsafe methods such as flushing down the toilet or throwing pills directly into the trash), or “sufficient knowledge” (safe and appropriate disposal).

#### Minimal Knowledge

“They don’t really talk about it. You hear a lot about like all the people dying and like how, you know, it’s like this unstoppable public health issue. But… I haven’t really heard of many good ways to dispose.”

#### Limited Knowledge

“I know about [opioid disposal] and I’ve seen it at the Smith Pharmacy or something.”

Interviewer: “What do you think about those programs?”

“I think it’s great. I mean, maybe for other pills.”

#### Sufficient Knowledge

“This hospital has always given me bags to get rid of my pain medication. It’s like a blue and white bag. It has powder in it, and you put your pills in and put water in and shake it and throw it away. Our sheriff’s department has a bin where you can drop-off used medications that we don’t need no more.”

Over half of participants (n = 29, 69%) said they would be more likely to dispose if it took less time (Table 4) and less likely to dispose if it took more time (Table 5). Similarly, more than half (n = 23, 55%) stated they would be more likely to dispose if they received a larger financial incentive. Financial incentives encouraged disposal through several mechanisms: compensation for inconvenience, reward for desired behavior, and alleviation of the perceived sunken cost of expensive prescriptions. Beyond convenience and financial incentive, nearly half of participants felt anticipation of pain would make them less likely to dispose (n = 20, 48%). Others felt nondisposal was justified because they had increasing difficulty getting opioids prescribed for their pain (n = 11, 26%) or wanted to use the leftover pills recreationally (n = 10, 24%). In contrast, participants were motivated to dispose to keep family members (particularly children) safe (n = 18, 43%) or by moral beliefs about community responsibility (n = 15, 36%). Concerns about the risk of addiction (n = 11, 26%), theft (n = 11, 26%), or environmental harm (n = 9, 21%) also motivated disposal.

**TABLE 4.** Facilitators to Opioid Disposal, With Exemplar Quotations

Facilitators (More Likely to Dispose)		
More convenient	29 (69%)	“It feels like anything at home, anything you can do at home, you would for sure do that for free, right? That little provided at-home-kit would be huge.”
		“Most of the time, I’m going to be most unlikely to dispose of the medication if it requires me to drive. I might consider the $50 one. But that’s all. The rest of them aren’t worth it to me...It takes time and it takes gas and I’m busy. I’m just not interested.”
Larger financial incentive	23 (55%)	“Five bucks, that’s not worth my time…ones and fives are like quarters and pennies nowadays. Twenty-five bucks, yeah, it’s worth my time.”
		“It’d depend on the day, because sometimes this money would come in mighty handy towards the end of the month.”
Family member safety	18 (43%)	“And the reason why I’d like to get rid of it is just because I’ve got six kids at home…I wouldn’t want one of my kids to get addicted to that stuff. I’ve seen too many people ruin lives with pills.”
		“You see stuff on the news that kids-- we don’t have any small children, but we had grandkids that we’re young and we didn’t need them get into that stuff.”
Moral belief	15 (36%)	“It’s like a public or a community service thing just to get rid of them.”
		“I don’t want to inflict nobody pain, sorrow or whatever. I don’t want to be the effect of somebody having passed out or blacked out or even killed. I just get rid of them.”
Risk of addiction	11 (26%)	“To be completely honest with you I personally, again, I’ve seen the negative side. So I know that the longer you hold on to them and then that’s very easy to transition to some sort of problem there.”
Risk of theft	11 (26%)	“I would want to get rid of it, because I don’t want anybody come looking for it … I live in an apartment complex, a low-income ousing apartment…[theft] is a possibility if you watch the news.”
Risk of harming environment through informal disposal	9 (21%)	“I don’t want them to go into the water source. You know? Especially in Utah, where you guys are relying on groundwater. So, you don’t want to throw them away. You don’t want to put them in the toilet.”

**TABLE 5.** Barriers to Opioid Disposal, With Exemplar Quotations

Barriers (Less Likely to Dispose)		
Less convenient	29 (69%)	“I just want to get rid of it, but it’s not really worth the effort to drive somewhere to do it… I live in a rural area…for me, this is a convenience thing.”
Smaller financial incentive	23 (55%)	“If they offered me money I’d probably dispose it. If they didn’t I probably wouldn’t. I would keep it, because I pay more than 50 bucks for my script. I mean, drugs are very expensive.”
Anticipation of future pain	20 (48%)	“If I had any opiate medicine left, it would literally sit in my dresser until something happened …I’d rather take that than go to the ER and get charged $5,000.”
		“Okay. So, the truth of the matter is if I have extra opioids, my tendency would be to keep them for another time when I might be really hurting… I’m not a drug addict, but I might like one in the drawer for another time if I needed one.”
Perception of opioid scarcity	11 (26%)	“I suffer from kidney stones. And it is probably the hardest thing in the world to get any opioids to get when I go into urgent care. … if I don’t have one or two around, it means the difference between agony and being able to just sleep through it. So I understand that there’s a need to control opioids, but I’m very safe with where I hide my opioids and I sort of depend on them. If it were easier to get them when I need them, it would be different.”
Desire to use opioids for recreation	10 (24%)	“I’m an addict. If I... have some left over, they’re worth more to me than the $50…I care more about the pills than money.”

## DISCUSSION

The current study examined how convenience (time required to dispose) and financial incentives influence patients’ willingness to dispose of leftover opioids after surgery. Disposal convenience was a more important factor than financial incentives, with time requirements of 15 minutes or less eliciting disposal rates over 50% regardless of financial incentives. As the time to dispose reached 30 minutes or more, increasing financial incentives were needed to encourage disposal. In qualitative interviews, participants identified anticipation of future pain, perception of opioid scarcity, and recreational use as additional barriers to disposal. Conversely, family safety, moral beliefs, risk of addiction, theft, and environmental harm through informal disposal were facilitators to disposal.

We found convenience drove willingness to dispose to a greater extent than financial incentives. Without financial incentive, 62% and 51% of participants were still willing to dispose when the time required was <5 or 15 minutes, respectively. Conversely, even with financial incentives of $50, only 36% of participants were willing to dispose if the time required was >60 minutes. These results suggest the 29% return rate of leftover opioids (when paid up to $50) reported by a Veterans Affairs pilot study is not anomalous and financial incentives alone are not enough to drive leftover opioid disposal.^26,27^ Our findings also may explain why most interventions providing home disposal bags to patients reported disposal rates of 55%–86%.^19–21^ Disposal bags allow patients to neutralize and dispose of leftover opioids at home with regular trash quickly and safely. Alternately, installing a convenient clinic drop box where surgery patients returned for follow-up visits (combined with phone reminders 1–3 days before their visit) led to a disposal rate of 83%.^7^

Despite the clear importance of convenience, financial incentives may still be an important facilitator to improving opioid disposal rates. Financial incentives have been used successfully in other health campaigns, including interventions promoting smoking abstinence^38^ and vaccination.^39^ In our study, financial incentives consistently increased disposal rates across disposal scenarios that required the same amount of time. Compared to scenarios with no financial incentives, a $50 incentive increased disposal rates by 21%–31%, depending on the time required to dispose. In our prior qualitative study of disposal in rural communities, some participants highlighted the cost (in time and money) to drive to far away disposal sites and the money spent on expensive prescriptions as barriers to disposal.^30^ Financial incentives may offset these expenses, particularly for low-income and rural patients who are disproportionately affected by the opioid crisis.^40^ While we did not find an association between disposal and household income or rural residency, our qualitative findings suggest targeted interventions using financial incentives could promote disposal by patients with low incomes or those in rural communities. Distribution of home disposal bags with or without a small financial incentive may be sufficient for some populations. For others, convenient disposal opportunities may not be sufficient without a financial incentive.

While convenience and financial incentives play a central role in patient decision-making about disposal, future disposal interventions will need to address other barriers. For example, a randomized trial comparing the distribution of a home disposal bag, a patient education handout on proper disposal, and no intervention found no difference between the 3 arms (14%, 11%, and 10% disposal rates, respectively).^22^ The authors hypothesized the passive distribution of bags and education without engaging patients likely contributed to low disposal rates. Other education-only interventions have shown no to only modest benefits.^20,41^ One explanation for these low rates is passive patient education is rarely effective. Patient education should be delivered through multiple modalities and the messaging should be reinforced through multiple sessions.^42,43^ Tailoring education to address individual concerns (eg, risk to children, addiction, theft, environmental harm) or moral beliefs could further motivate disposal. For example, parents are more motivated to dispose after receiving messaging about the risks of undisposed opioids to children.^44^ Elderly patients may be more likely to dispose when educated about the risks of delirium, adverse drug interactions, falls, and/or fractures.^45^ Most educational efforts focus on raising awareness about the importance of opioid disposal. However, raising awareness is only the first in a series of steps patients undertake toward disposal. Integrating the barriers and facilitators identified in this study and others into behavior change frameworks such as the Precaution Adoption Process Model^30^ or Information-Motivation-Behavioral Skills Model^46^ can guide both the development and evaluation of future interventions. For example, both models emphasize the importance of not only addressing awareness but also patient motivations, indecision, behavioral skills, and the conversion of intention into action.

Our study has several limitations. First, our study was conducted at a single academic institution, which limits the generalizability of our findings. Our population spanned the rural-urban divide and represented a range of household incomes, but perceptions of the risks of opioids likely differ across communities, regions, and cultures.^31^ Specifically, low-income (income <$50,000) and high-income (>$100,000) participants were underrepresented in our study population (31% vs 38% nationally and 27% vs 34% nationally), as were non-White (22% vs 38% nationally) and rural (31% vs 19.3% nationally) patients.^47^ The effect of financial incentives on disposal rates is likely to differ in populations at the extremes of wealth and the need to promote convenience is likely reduced for urban populations with improved access to more conventional methods of opioid disposal. Unique populations may have different barriers and facilitators driving patients’ disposal willingness. The effects of convenience and financial incentives on promoting opioid disposal are likely maintained across populations; however, the interventions required to provide enough incentive to motivate people to dispose may differ for different populations. Further studies in different communities could help validate our findings and help identify other factors associated with enhancing opioid disposal. Second, our study presented participants with a hypothetical disposal situation and actual behaviors may differ. Social desirability bias may have led to more participants being willing to dispose than they would in real situations. However, the openness of our participants’, particularly those who expressed a desire to use leftover opioids recreationally or for larger financial incentives, suggests willingness to honestly report actions in a confidential setting. Also, the wide variation found in our study across the scenarios suggests that convenience and financial incentives do influence willingness to dispose, but real-world testing of these scenarios is needed. Another limitation is that we chose to use an inductive analysis without a theoretical framework. The strength of this approach is that participants themselves defined potential barriers and facilitators in the context of financial incentives and convenience. However, participants may not include barriers they did not think of which might be otherwise prompted if we had used a framework such as the socioecological model (eg, public policies). Finally, we focused on convenience and financial incentives because prior studies had identified these as major barriers and facilitators to disposal. But the effect of other factors on disposal willingness (identified in our qualitative interviews and other studies) will need to be tested further.

## CONCLUSIONS

Timely interventions promoting safe and appropriate disposal of leftover prescription opioids are needed to reduce opioid-related harms and deaths. Interventions should prioritize convenience to elicit the highest disposal rates. While time matters more than money to most patients, financial incentives may boost disposal rates for some populations. Designing interventions targeted at individual patients could further increase disposal and prevent many of the harms caused by excess prescription opioids.

## ACKNOWLEDGMENTS

The authors thank Drs T. Bartley Pickron, Luke Martin, and Courtney L. Scaife for allowing our research team to interview participants in their surgery clinics.

P.D. and J.B. participated in research study design, data collection, qualitative analysis, quantitative statistical analysis, and drafting the article. J.K.K. participated in data collection and qualitative analysis. E.L.S. provided major revisions and editing of the article. G.J.S. participated in research study design and quantitative statistical analysis. J.E.J. participated in research study design and qualitative analysis. J.N.C. provided critical interpretation of the qualitative and quantitative analysis. K.A.K. participated in research study design and qualitative analysis and provided critical interpretation of the qualitative and quantitative analysis and major revisions and editing of the article. A.H.S.H. participated in research study design and provided critical interpretation of the qualitative and quantitative analysis and major revisions and editing of the article. L.C.H. participated in research study design, qualitative analysis, and quantitative statistical analysis and provided major revisions and editing of the article.

## Supplementary Material


