# Supplementary material for: Patient Willingness to Dispose of Leftover Opioids After Surgery: A Mixed Methods Study
Source: Ann Surg Open. 2022 Dec 7;3(4):e223. doi: 10.1097/AS9.0000000000000223 (PMC9780041; doi:10.1097/AS9.0000000000000223)
Supplement: Supplementary file 1 [file as9-3-e223-s001.pdf]

INTRODUCTION

My name is [*interviewer*]. Thank you again for agreeing to participate in today's study. The study will take about 15 minutes. My role will be to ask questions and listen to your responses.

How do you wish to be called [Mr. Smith/Ms. Jones]…..? While I know this name, I want to make sure that you know that your real name will not be known to anyone outside the study team.

This conversation is completely confidential, and your responses will be used only for research purposes. We will remove any personal, identifying information in the interviews during the transcription process, and no identifiable information will be shared with your medical team. None of the information you share can be used against you, it will not be incorporated into your medical chart, and it will it not affect your treatment in any way.

This interview will be audio recorded. The recordings will be heard only by the research staff on this project, or by a professional transcriptionist.

We are interested in learning more about how likely you would act in a hypothetical situation after surgery.

There are no right or wrong answers to any of these questions. As we go through this study, please feel free to think aloud. I want to hear your thoughts and experiences.

Do you have any questions for me before we begin?

MATRIX COMPLETION

Imagine you've undergone a major operation. You were prescribed opioids for pain and your pain has resolved. You have some left-over opioids at home. With this situation in mind, complete the matrix below. Each column represents a way you can safely dispose of your unused opioids. Each row represents the amount of money you will receive for disposing of your opioids.

For example, this top left box represents a scenario in which you dispose of your opioids for free, and it takes you less that 5 minutes at home with a provided disposal kit. And this bottom right box represents a scenario in which you receive $50 to dispose of your opiates, and it takes you 60 minutes to drive to a locked drop box at a pharmacy.

Please fill in each box with a number 1 to 5 to represent how likely you are to dispose of your unused opioids in the given scenario. A score of **1** means you are '**very unlikely'** to dispose in the scenario. **2** means you are '**unlikely**' to dispose. **3** is **neutral**: you are equally likely to dispose or not dispose. **4** means you are '**likely'** to dispose, and **5** means you are **'very likely**' to dispose. I've listed the rating system here [point to top of page with matrix]. Only whole numbers are allowed, please do not respond with ½ numbers.

**Begin recorder*.

Today's date is 00/00/0000. This is [interviewer's name], and I am speaking with participant [ID #]. For the record, do you consent to participate in today's interview and be recorded?

	<5 minutes with a provided home disposal kit	*15 minutes to drive to a drop box*	*30 minutes to drive to a drop box*	*60 minutes to drive to a drop box*
free				
$5				
$25				
$50				

For your reference, possible responses:

1=**very unlikely** to dispose
2=**unlikely** to dispose
3=**neutral**: equally likely to dispose and not dispose
4=**likely** to dispose
5=**very likely** to dispose

SEMI-STRUCTURED INTERVIEW QUESTIONS AND PROBES

I'd like to ask you a few questions. As I mentioned earlier, this portion will be audio recorded and will not be shared with any one beyond the research staff and professional transcriptionist for this project.

What do you know about disposal of left-over opioids?

Could you walk me through your decision-making process as you were completing the matrix above?

In general, Is the financial incentive or the convenience more important to you?

> [Now, probe interviewer on why they selected certain numbers. For example…]
>
> I noticed that you rated this box, 60 minutes and $50 an X. Why did you do that? How much money would it take you to dispose in that hypothetical situation. How much if it took you 30 minutes? Why do you say $XXX?

What other factors would make you **more likely** to dispose?

What other factors would make **you less likely** to dispose?

DEMOGRAPHICS

Thank you so much for sharing your thoughts. In order to not access any of your clinical records for this study, I need to ask you a few more questions. This will not be linked to the medical chart in any way.

1. How old are you? _______

2. How would you describe your gender?

- Male
- Female
- Non-binary
- Other: ________________
- Prefer not to say

3. Do you consider yourself…? (Feel free to select more than one)

- White
- Black or African-American
- American Indian or Alaska Native
- Asian
- Native Hawaiian or other Pacific Islander
- Other: ____
- Prefer not to say

4. Do you identify as Hispanic or Latino/a?

- Yes
- No
- Prefer not to say

5. Have you ever filled an opioid prescription in the past?

- Yes
- No

6. Have you filled an opioid prescription in the past year?

- Yes
- No

7. Have you used opioids regularly in the past year? (defined as >1x per month for several months consecutively)

- Yes
- No

8. If you had unused opioids from this prescription, what did you do with them?

9. Do you know anybody who abuses opioids?

- Yes
- No

10. What category most closely resembles your yearly household income:

- $0-25,000
- $25,001-50,000
- $50,001-75,000
- $75,001-100,000
- $100,001-125,000
- $125,001-150,000
- >$150,000

11. What is your home zip code?

12. Do you have regular access to a private vehicle?

- Yes
- No

13. Do you currently smoke, use tobacco products, or vape (e.g. e-cigarettes)?

- Yes
- No

14. Do you have a personal history of

(Please check “Yes” or “No” for each condition listed)

Fibromyalgia	Yes	No
Chronic pain	Yes	No
Headaches/migraines	Yes	No
Sexual abuse	Yes	No
Depression	Yes	No
Anxiety	Yes	No
Attention deficit disorder	Yes	No
Obsessive compulsive disorder	Yes	No
Schizophrenia	Yes	No
Alcohol abuse	Yes	No
Opioid abuse	Yes	No
Recreational drug use	Yes	No

COREQ GUIDELINES

1. **Interviewer/facilitator:** Phoebe Draper, Jaqueline Kobayashi
2. **Credentials:** BA, BS
3. **Occupation:** Medical students
4. **Gender:** Female
5. **Experience and training:** Prior experience with qualitative methodology and coding through prior research. Supervision and training from trained experts in qualitative methodology.
6. **Relationship established with participants:** Collegial relationship. Interviewers had no prior relationship with participants and were not involved with participants' clinical care teams.
7. **Participant knowledge of the interview:** Interviewers introduced themselves with a brief introduction, including mentioning their credentials as medical student and informing participants that the interviewers were not a part of the participants' clinical care teams.
8. **Interviewer characteristics:** Both interviewers are medical students interested in opioid stewardship in the perioperative period.
9. **Methodological orientation and theory:** Thematic content analysis
10. **Sampling:** Purposeful sampling - adult patients (age > 18 years) who presented to general surgery clinics for preoperative consultation at a single academic medical center between 1/28/2021-3/29/2021 were invited to participate.
11. **Method of approach:** Face-to-face and Zoom video chat
12. **Sample size:** 42
13. **Non-participation:** 10% of invited participants. Participants were not included secondary to refusal, typically due to time constraints
14. **Setting of data collection:** A private room at a general surgery clinic office
15. **Presence of non-participants:** No. Interviews were limited to the participant and interviewer
16. **Description of sample:** The study population is described in the manuscript in Table 1. Median age was 53, 53% were female, 78% were white, and most had a household income of $50,000-$100,000 (38%). Please see Table 1 for further details
17. **Interview guide:** Interview guide was created with assistance from experts in surgery, opioid stewardship, rural health, and qualitative research. The interview guide was pilot tested with potential study participants prior to data collection, with modification of the interview guide as needed.
18. **Repeat interviews:** None
19. **Audio/visual recording:** Audio recording only
20. **Field notes:** None
21. **Duration:** Interviews averaged 15-20 minutes, and ranged in length from 10-30 minutes.
22. **Data saturation:** Thematic saturation was achieved for all interview questions
23. **Transcripts returned:** Transcripts were not returned to participants for comment and/or correction.
24. **Number of data coders:** 2
25. **Description of coding tree:** The final codebook consisted of 19 unique themes spread across 4 domains: financial incentives, convenience, other facilitators, and other barriers.
26. **Derivation of themes:** Themes were summarized based on the final codebook.
27. **Software:** Microsoft Excel
28. **Participant checking:** Final results were not shared with participants to provide feedback on the findings.
29. **Quotations presented:** Yes. Exemplary quotations that illustrate the key themes are presented in the manuscript.

30. **Data and findings consistent:** There was a high level of concordance between the qualitative data and presented findings, including the findings identified in the quantitative arm of this study.
31. **Clarity of major themes:** Participants were more likely to dispose of leftover opioids if it was more convenient to dispose and they received a larger financial incentive. Financial incentives encouraged disposal through several mechanisms: compensation for inconvenience, reward for desired behavior, and alleviation of the perceived sunken cost of expensive prescriptions.
32. **Clarity of minor themes:** Nearly half of participants felt anticipation of pain would make them less likely to dispose (n=20, 48%). Others felt non-disposal was justified because they had increasing difficulty getting opioids prescribed for their pain (n=11, 26%) or wanted to use the leftover pills recreationally (n=10, 24%). In contrast, participants were motivated to dispose to keep family members (particularly children) safe (n=18, 43%) or by moral beliefs about community responsibility (n=15, 36%). Concerns about the risk of addiction (n=11, 26%), theft (n=11, 26%), or environmental harm (n=9, 21%) also motivated disposal.
